# Chronic Exposure to Combined Carcinogens Enhances Breast Cell Carcinogenesis with Mesenchymal and Stem-Like Cell Properties

**DOI:** 10.1371/journal.pone.0108698

**Published:** 2014-11-05

**Authors:** Lenora Ann Pluchino, Hwa-Chain Robert Wang

**Affiliations:** 1 Department of Biomedical and Diagnostic Sciences, College of Veterinary Medicine, University of Tennessee, Knoxville, Tennessee, United States of America; 2 Graduate School of Genome Science and Technology, University of Tennessee, Knoxville, Tennessee, United States of America; National University of Ireland Galway, Ireland

## Abstract

Breast cancer is the most common type of cancer affecting women in North America and Europe. More than 85% of breast cancers are sporadic and attributable to long-term exposure to small quantities of multiple carcinogens. To understand how multiple carcinogens act together to induce cellular carcinogenesis, we studied the activity of environmental carcinogens 4-(methylnitrosamino)-1-(3-pyridyl)-1-butanone (NNK) and benzo[a]pyrene (B[a]P), and dietary carcinogen 2-amino-1-methyl-6-phenylimidazo[4,5-b]pyridine (PhIP) using our breast cell carcinogenesis model. Our study revealed, for the first time, that combined NNK and B[a]P enhanced breast cell carcinogenesis chronically induced by PhIP in both non-cancerous and cancerous breast cells. Co-exposure was more potent than sequential exposure to combined NNK and B[a]P followed by PhIP in inducing carcinogenesis. Initiation of carcinogenesis was measured by transient endpoints induced in a single exposure, while progression of carcinogenesis was measured by acquisition of constitutive endpoints in cumulative exposures. Transient endpoints included DNA damage, Ras-Erk-Nox pathway activation, reactive oxygen species elevation, and increased cellular proliferation. Constitutive endpoints included various cancer-associated properties and signaling modulators, as well as enrichment of cancer stem-like cell population and activation of the epithelial-to-mesenchymal transition program. Using transient and constitutive endpoints as targets, we detected that a combination of the green tea catechins ECG and EGCG, at non-cytotoxic levels, was more effective than individual agents in intervention of cellular carcinogenesis induced by combined NNK, B[a]P, and PhIP. Thus, use of combined ECG and EGCG should be seriously considered for early intervention of breast cell carcinogenesis associated with long-term exposure to environmental and dietary carcinogens.

## Introduction

Breast cancer is the most common type of cancer and second leading cause of cancer-related death among women in North America and Europe [1], [2]. Over 85% of breast cancers occur sporadically due to long-term exposure to low doses of multiple carcinogens [3]–[7]. Thus, it is important to investigate how multiple carcinogens act together to induce cellular carcinogenesis. We have developed a cellular model that mimics breast cell carcinogenesis induced by cumulative exposures to physiologically-achievable doses of environmental and dietary carcinogens to understand the cellular, biochemical, and molecular changes involved in cellular carcinogenesis for the purposes of intervention.

American lifestyles involve frequent consumption of high-temperature cooked meats containing carcinogens, such as 2-amino-1-methyl-6-phenylimidazo[4,5-b]pyridine (PhIP), and wide exposures to smoke and polluted air containing 4-(methylnitrosamino)-1-(3-pyridyl)-1-butanone (NNK) and benzo[a]pyrene (B[a]P). PhIP is the most abundant heterocyclic amine found in meat cooked at high temperatures, and consumption of PhIP at microgram levels results in systemic exposure at low nanomolar levels [8], [9]. Gastric administration of PhIP induces mammary tumors in rats [10], [11], and epidemiological studies have indicated a close association between well-done meat consumption and human breast cancer risk [12]–[14]. NNK, a tobacco-specific nitrosamine ketone, can be detected at picomolar concentrations in body fluids of tobacco users [15]–[17]. Although gastric administration of NNK into rats resulted in DNA adducts and tumor development in the mammary gland [18], [19], NNK is not yet recognized as a mammary carcinogen. The link between smoking and breast cancer is controversial; however, recent studies indicate that exposure to tobacco smoke can increase breast cancer risk, especially in post-menopausal women [20]–[22]. Thus, the role of tobacco carcinogens in breast cancer needs to be clarified. B[a]P, on the other hand, is recognized as a weak mammary carcinogen. B[a]P is a polycyclic aromatic hydrocarbon present in carbon exhaust, charcoal-barbequed foods, and tobacco smoke; it can be found in picomolar concentrations in human fat and liver [23]–[28].

Our studies have shown that NNK at100 pmol/L, B[a]P at 100 pmol/L, and PhIP at 10 nmol/L are able to induce initiation and progression of breast cell carcinogenesis [29]–[35]. A single exposure to these carcinogens induces transient changes, which play essential roles in induction of carcinogenesis and can be used as transient endpoints to promptly reveal carcinogenic activity. Cumulative exposures to carcinogens progressively induce cellular acquisition of various cancer-associated properties and activation of associated pathways; these properties are measurable constitutive endpoints used to determine the progression of cellular carcinogenesis from non-cancerous to pre-cancerous and cancerous stages [29]–[35]. Our model also reveals increases of cancer stem-like cell populations and activation of the epithelial-to-mesenchymal transition (EMT) program during carcinogen-induced cellular carcinogenesis [35], [36]. Development of cancer stem-like cells, involving induction of the EMT program, plays important roles in generating and maintaining pre-malignant and malignant lesions [37]. Thus, we also used increased cancer stem-like cell population and induced EMT program as constitutive endpoints in our studies. We then used these endpoints as targets to identify preventive agents, such as green tea catechins (GTCs) epicatechin (EC), epicatechin-3-gallate (ECG), epigallocatechin (EGC), and epigallocatechin-3-gallate (EGCG), at non-cytotoxic levels, capable of intervening in breast cell carcinogenesis induced by NNK, B[a]P, or PhIP [31]–[36].

In this communication, we report the potency of co-exposure versus pre-exposure of combined NNK and B[a]P (NB) with PhIP in chronic induction of breast cell carcinogenesis. Co-exposure to NB and PhIP (NBP) induced higher levels of transient and constitutive endpoints than pre-exposure to NB followed by PhIP. We also studied the activity of ECG and EGCG, at non-cytotoxic levels, in suppression of NBP-induced endpoints. We determined that a combination of ECG and EGCG was more effective than either agent alone, at equivalent doses, in intervention of NBP-induced cellular carcinogenesis.

## Materials and Methods

### Cell cultures and reagents

MCF10A (American Type Culture Collection [ATCC], Rockville, MD) and derived cell lines were maintained in complete (CM) medium (1∶1 mixture of DMEM and HAM's F12, supplemented with 100 ng/mL cholera enterotoxin, 10 µg/mL insulin, 0.5 µg/mL hydrocortisol, 20 ng/mL epidermal growth factor, and 5% horse serum) [29]–[36]. Human breast cancer MCF7 cells (ATCC) were maintained in DMEM supplemented with 10% heat-inactivated fetal calf serum [34], [35]. All cultures were supplemented with 100 U/mL penicillin and 100 µg/mL streptomycin and maintained in 5% CO_2_ at 37°C. Stock solutions of NNK (Chemsyn, Lenexa, KS), B[a]P (Aldrich, Milwaukee, WI), PhIP (Midwest; NCI Chemical Carcinogen Reference Standard Repository), U0126 (Cell Signaling, Beverly, MA), and CM-H_2_DCF-DA (Invitrogen, Carlsbad, CA) were prepared in DMSO; ECG, EGCG (Sigma-Aldrich, St. Louis, MO), and *N*-acetyl-L-cysteine (NAC) (Alexis, San Diego, CA) were prepared in distilled water. All were diluted in CM medium for assays.

### Chronic induction of cellular carcinogenesis

Twenty-four h after each subculturing, human breast cells were treated with the indicated carcinogens for 48 h as one cycle of exposure for 20 cycles. Cells were subcultured every 3 days [29]–[36].

### DNA Damage

DNA damage was detected with a comet assay [38]. A total of 2×10^4^ cells in ice-cold phosphate-buffered saline (PBS) were mixed with 1% low-melting agarose (Fisher, Fair Lawn, NJ) and placed on agarose-coated slides. Slides were immersed in lysis solution, electrophoresed, stained with propidium iodide and examined with a fluorescence microscope (Carl Zeiss Inc., Thornwood, NY) as described previously [34], [35]. Fifty nuclei per slide were scored for tail moment (% of DNA in the tail X tail length) using CometScore software (Tritek, Sumerduck, VA).

### Reduced dependence on growth factors

A total of 5 × 10^3^ cells were seeded in 60-mm culture dishes and maintained in low-mitogen (LM) medium (1∶1 mixture of DMEM/F12, 2 ng/mL cholera enterotoxin, 200 ng/mL insulin, 10 ng/mL hydrocortisol, 0.4 ng/mL epidermal growth factor, and 0.1% horse serum), containing reduced total serum and mitogenic additives to 2% of the concentration formulated in CM medium, for 10 days to develop cell colonies [32]–[35].

### Anchorage-independent growth

A total of 1×10^4^ cells were mixed with soft agar consisting of 0.4% low-melting agarose (Sigma-Aldrich) in a mixture (1∶1) of CM medium with 3-day conditioned medium prepared from MCF10A cultures, plated on top of a 2% low-melting agarose base layer in 60-mm culture dishes and maintained for 14 days to develop cell clones [32]–[35].

### Measurement of intracellular ROS

Cells were incubated with 5 µmol/L CM-H_2_DCF-DA for 1 h to detect ROS by flow cytometry as described previously [32]–[35]. Mean fluorescence intensity of dichlorodihydrofluorescein (DCF) was quantified using Multicycle software (Phoenix Flow System, San Diego, CA).

### Cellular proliferation

Cellular proliferation was determined using the 5-bromo-2-deoxyuridine (BrdU) cell proliferation enzyme-linked immunosorbent assay (ELISA) kit (Roche, Indianapolis, IN); BrdU-labeled cells were quantified using an ELISA reader (Bio-Tek, Winooski, VT), as performed previously [32]–[35].

### Reverse transcription PCR

Total RNA isolated from cultures using the Absolutely RNA kit (Stratagene, La Jolla, CA) was reverse transcribed to complementary DNA using the Verso cDNA Kit (Thermo Scientific, Waltham, MA). cDNAs were subjected to PCR for H-Ras (forward: 5′-GACGGAATATAAGCTGGTGG-3′; reverse: 5′-AGGCACGTCTCCCCATCAAT-3′) and β-actin (forward: 5′-GGACTTCGAGCAAGAGATGG-3′; reverse: 5′-AGCACTGTGTTGGCGTACG-3′). PCR products were electrophoresed on agarose gels and visualized using ethidium bromide staining.

### Immunoblotting

Cell lysates were resolved by electrophoresis in 10% or 12% SDS–polyacrylamide gels and transferred to nitrocellulose filters for immunoblotting, as described previously [32]–[35], with antibodies specific to H-Ras, phosphorylated Erk1/2 (p-Erk1/2), Erk1/2, Nox-1, EpCAM, E-cadherin, MMP-9, Vimentin, and β-actin (Santa Cruz Biotechnology, Santa Cruz, CA). Antigen-antibody complexes were detected by Supersignal West Dura chemiluminescence kit (Pierce, Rockford, IL).

### Serum-independent non-adherent growth

A total of 1×10^4^ cells were seeded on top of 1% agarose-coated, non-adherent 100-mm culture plates, incubated in serum-free CM supplemented with 0.4% bovine serum albumin and maintained for 10 days to develop mammospheres [35], [36].

### Aldehyde dehydrogenase (ALDH) assay

An ALDEFLUOR Kit (StemCell Technologies, Durham, NC) was used to detect ALDH-positive cells. A total of 1×10^5^ cells were mixed with activated Aldefluor substrate BODIPY-aminoacetaldehyde (BAAA) and incubated in the presence and absence of the ALDH inhibitor diethylaminobenzaldehyde (DEAB), followed by flow cytometric analysis as described previously [35], [36]. Mean fluorescence intensity of cells was quantified using Multicycle software (Phoenix Flow System, San Diego, CA). Cells incubated with BAAA in the presence of DEAB were used to establish baseline fluorescence for determining the ALDH-positive cell population in which ALDH activity was not inhibited by DEAB.

### *In vitro* cell invasion and migration

The cell invasion assay was performed using 24-well transwell insert chambers with a polycarbonate filter with a pore size of 8.0 µm (Costar, Corning, NY). A total of 2×10^4^ cells in serum-free medium were seeded on top of a Matrigel-coated filter (BD Biosciences, Franklin Lakes, NJ) in each insert chamber. Then, the insert chambers were placed into wells on top of culture medium containing 10% horse serum as a chemoattractant. The migration assay was performed using 24-well transwell insert chambers with a polycarbonate filter without Matrigel. The invasive or migratory ability of cells was determined by the number of cells translocated to the lower side of filters [35], [36].

### Cell motility wound-healing assay

Cells were seeded in 6-well plates and grown to confluence in CM medium. Cells were rinsed with PBS and serum-starved for 15 h in DMEM/Ham’s F12 medium containing 2% horse serum. The monolayer was then scratched with a 23-gauge needle (BD Biosciences) to generate wounds, rinsed with CM medium to remove floating cells, and maintained in CM medium. The wounded areas were examined 6, 12, and 24 h after scratches to detect healing. The area not healed by the cells was subtracted from the total area of the initial wound to calculate the wound healing area using Total Lab TL100 software (Total Lab, Newcastle, NE).

### Statistical analysis

The Student *t* test was used to analyze statistical significance, indicated by * *P*<0.05, ** *P*<0.01, and *** *P*<0.001; a *P* value <0.05 was considered significant.

## Results and Discussion

### Enhanced cellular acquisition of cancer-associated properties by combined carcinogens

**Figure 1:**
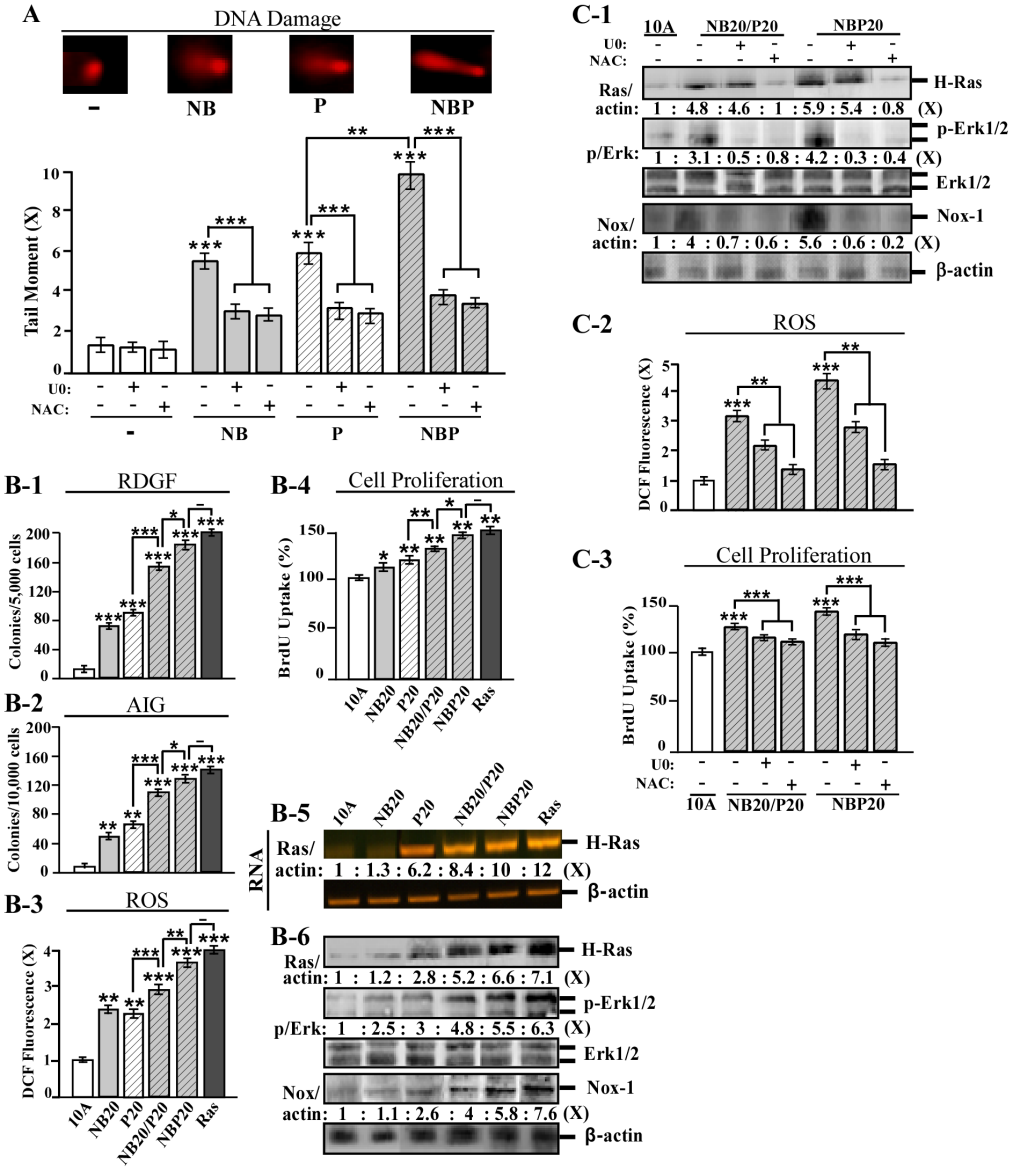
Enhanced cellular acquisition of cancer-associated properties by combined carcinogens. (A) MCF10A cells were treated with combined 100 pmol/L NNK and 100 pmol/L B[a]P (NB), 10 nmol/L PhIP (P), or combined NB and PhIP (NBP) in the absence and presence of 10 µmol/L U0126 (U0) or 5 mmol/L NAC for 24 h. DNA damage was measured by a comet assay and normalized by the value of average tail moment determined in untreated counterpart cells, set as 1 (X, arbitrary unit). Representative images detected in the comet assay are shown. (B-1 to B-6) MCF10A (10A) cells were repeatedly exposed to NB, PhIP, or NBP for 20 cycles, resulting in the NB20, P20, and NBP20 cell lines, respectively. NB20 cells were then exposed to PhIP for an additional 20 cycles resulting in the NB20/P20 cell line. MCF10A-Ras (Ras) cells were used as a malignant control. (C-1 to C-3) NB20/P20 and NBP20 cells were treated with 10 µmol/L U0 or 5 mmol/L NAC for 48 h. (B-1) To determine cellular acquisition of RDGF, cells were maintained in LM medium for 10 days. Cell colonies ≥0.5 mm diameter were counted. (B-2) To determine cellular acquisition of AIG, cells were seeded in soft agar for 14 days. Cell colonies ≥0.1 mm diameter were counted. (B-3 and C-2) Relative level of ROS as fold induction (X, arbitrary unit) was normalized by the level determined in untreated cells, set as 1. (B-4 and C-3) Relative cell proliferation was determined and normalized by the value of BrdU detected in untreated cells, set as 100%. (B-5) Total RNA was isolated and analyzed by RT-PCR with specific primers to determine relative gene expression levels of H-Ras, with β-actin as a control, and these levels were quantified by densitometry. (B-6 and C-1) Cell lysates were analyzed by immunoblotting using specific antibodies to detect levels of H-Ras, phosphorylated-Erk1/2 (p-Erk1/2), Erk1/2, and Nox-1, with β-actin as a control, and these levels were quantified by densitometry. The levels of H-Ras (Ras/actin) and Nox-1 (Nox/actin) were calculated by normalizing with the level of β-actin and the level set in untreated control cells as 1 (X, arbitrary unit). Levels of specific phosphorylation of Erk1/2 (p/Erk) were calculated by normalizing the levels of p-Erk1/2 with the levels of Erk1/2, then the level set in control cells as 1 (X, arbitrary unit). Columns, mean of triplicates; bars, SD. All results are representative of three independent experiments. Statistical significance is indicated by * *P*<0.05, ** *P*<0.01, and *** *P*<0.001.

Previous studies have shown that NNK and B[a]P exhibit comparable abilities to induce breast cell carcinogenesis [33], [34]. Combining NNK and B[a]P (NB) additively increases degrees of all acquired constitutive cellular endpoints except tumorigenicity [33], [34], indicating that NNK and B[a]P may act as weak breast carcinogens. We first used a comet assay to measure the ability of these carcinogens to induce cellular DNA damage after one exposure, which is essential for the induction of cellular carcinogenesis [37]. Exposure to combined NB and PhIP (NBP) induced a significantly higher level of DNA damage than either NB or PhIP (Figure 1A). Blockage of the ERK pathway (with the Mek-specific inhibitor U0126) or ROS production (with the general antioxidant NAC) significantly reduced NB-, PhIP-, and NBP-induced DNA damage (Figure 1A). These results indicate that co-exposure to NB and PhIP was more potent than either was alone to induce DNA damage for induction of cellular carcinogenesis, and the ERK pathway and ROS production are required for this process.

To verify whether cumulative exposures to NB and PhIP would result in enhanced cellular carcinogenesis, we exposed MCF10A cells to NB, PhIP, and NBP for 20 cycles, resulting in the NB20, P20, and NBP20 cell lines, respectively. NB20 cells were then exposed to PhIP for an additional 20 cycles, resulting in the NB20/P20 cell line. We then compared the potency of these resulting cell lines by measuring degrees of acquired cancer-associated properties/constitutive endpoints [29]–[35]. Growth factors and cellular adhesion to the extracellular matrix are required for normal epithelial cell survival; in contrast, cancerous cells acquire a reduced dependence on growth factors (RDGF) and anchorage-independent growth (AIG) to increase survivability [37]. Increased ROS production and cellular proliferation are also closely associated with cellular transformation [37]. We detected that NB20/P20 and NBP20 cells acquired higher degrees of RDGF (Figure 1B-1), AIG (B-2), ROS content (B-3), and cellular proliferation (B-4) than NB20 or P20 cells. NBP20 cells acquired higher degrees of these endpoints than NB20/P20 cells, reaching levels comparable to malignant control MCF10A-Ras cells, in which oncogenic H-Ras is ectopically expressed in MCF10A cells [39]. The results indicate that pre-exposure (NB20/P20) or co-exposure (NBP20) to NB significantly enhanced PhIP-induced constitutive endpoints, with co-exposure being the most potent.

Our previous studies showed that up-regulated H-Ras gene expression, as a constitutive molecular endpoint, and activated Ras-Erk-Nox pathway, as a constitutive biochemical endpoint, are essential for maintaining constitutive cellular endpoints induced by PhIP [35]. In studying if these molecular and biochemical changes were enhanced by NBP, we detected that cumulative pre- and co-exposures to NB and PhIP resulted in highly up-regulated H-Ras gene expression (Figure 1B-5), increased H-Ras protein level, and activated downstream Erk-Nox pathway (1B-6) in NB20/P20 and NBP20 cells; these endpoints were up-regulated to higher levels in NBP20 cells than in NB20/P20 cells but did not reach their counterpart levels in MCF10A-Ras cells. These results indicate that NNK and B[a]P can enhance the ability of PhIP to induce breast epithelial cell carcinogenesis, even though NNK is not considered a mammary carcinogen, and B[a]P is considered a weak mammary carcinogen. Co-exposure to these carcinogens is more potent than pre-exposure to induce cellular acquisition of cancer-associated properties. The constitutive biochemical endpoints of Ras-Erk-Nox pathway activation and ROS elevation were induced consistently with constitutive cellular endpoints and may play important roles in maintaining cancer-associated properties in NBP-exposed cells.

To verify roles the Erk pathway and ROS may play in NBP-exposed cells, we used U0126 to inhibit Mek and NAC to inhibit ROS in NB20/P20 and NBP20 cells. Treatment with U0126 did not affect H-Ras expression but reduced Erk activity, Nox-1 expression (Figure 1C-1), ROS level (1C-2), and cell proliferation (1C-3), indicating that the Erk-Nox pathway plays an important role in maintaining ROS production and cell proliferation in both NB20/P20 and NBP20 cells. NAC treatment suppressed the Ras-Erk-Nox pathway (Figure 1C-1), ROS level (1C-2), and cell proliferation (1C-3), indicating an essential role for ROS production in maintaining activated Ras-Erk-Nox pathway and increased cell proliferation. The results reveal a cross-talk between the Ras-Erk-Nox pathway and ROS production, which are necessary for maintaining increased cell proliferation, and possibly other cancer-associated properties, in NBP-exposed cells.

### Enhanced acquisition of stem-like and mesenchymal cell properties by combined carcinogens

**Figure 2:**
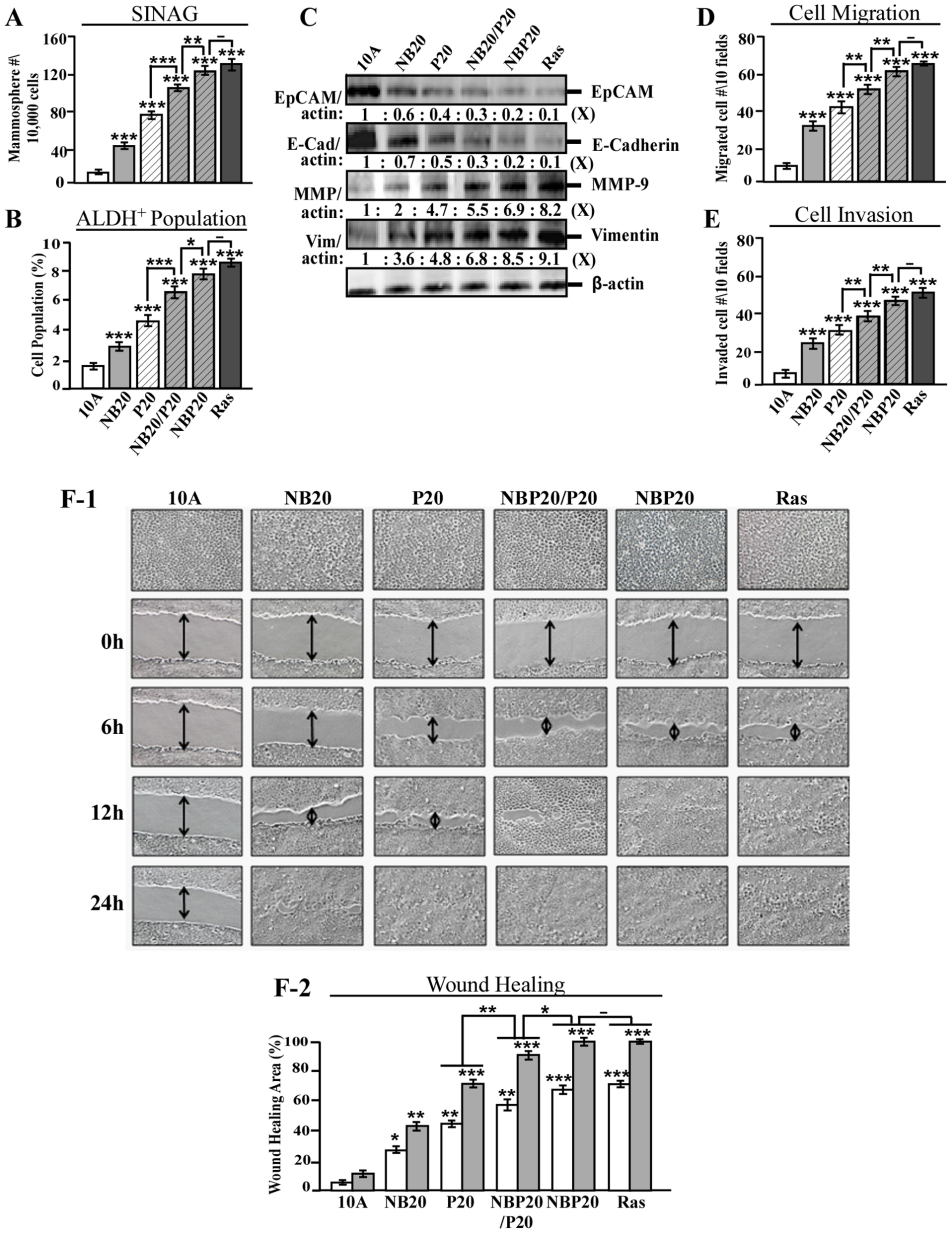
Enhanced acquisition of mesenchymal and stem-like cell properties by combined carcinogens. (A) To determine cellular acquisition of the ability of serum-independent non-adherent growth (SINAG), MCF10A (10A), NB20, P20, NB20/P20, NBP20, and MCF10A-Ras (Ras) cells were seeded in non-adherent cultures for 10 days; then, mammospheres (≥0.1 mm diameter) were counted. (B) Mammospheres were collected and trypsinized, and ALDH-expressing (ALDH^+^) cell population (%) was measured by flow cytometry. (C) Cell lysates were analyzed by immunoblotting using specific antibodies to detect levels of EpCAM, E-cadherin, MMP-9 and Vimentin, with β-actin as a control, and these levels were quantified by densitometry. The levels of EpCAM, E-cadherin, MMP-9, and Vimentin were calculated by normalizing with the level of β-actin and the level set in untreated control cells as 1 (X, arbitrary unit). (D) Cellular migratory and (E) invasive activities were determined by counting the numbers of cells translocated through a polycarbonate filter without or with coated Matrigel, respectively, in 10 arbitrary visual fields. (F-1) Cellular acquisition of increased motility was determined by wound healing assay. The wounded areas were examined (magnification, 100×) 6, 12, and 24 h afterward. Arrows indicate width of wounded areas. (F-2) To quantitatively measure cell motility detected in F-1, the area not healed by the cells was subtracted from the total area of the initial wound to calculate the wound healing area (%) at intervals of 6 (white columns) and 12 h (gray columns). Columns, mean of triplicates; bars, SD. All results are representative of three independent experiments. Statistical significance is indicated by * *P*<0.05, ** *P*<0.01, and *** *P*<0.001.

Mammary stem-like cells are able to self-renew in serum-free media, are known to exhibit high levels of ALDH activity, and have a unique ability to form discrete spheroid clusters called mammospheres in non-adherent cultures [40], [41]. We have reported that enrichment of ALDH-positive cell populations and increased formation of mammospheres were induced by cumulative exposures to NB [36] or PhIP [35] in MCF10A cells. However, increased stem-like cell population is not yet fully appreciated as a breast cancer-associated property. Growth of mammospheres (serum-independent non-adherent growth, SINAG) involves the properties of RDGF and AIG, both of which are enhanced by NBP exposure. Thus, it was important to understand whether NBP-enhanced cellular carcinogenesis was accompanied by enhanced stem-like cell properties. As shown in Figure 2, mammospheres (2A) and ALDH-positive cell population (2B) were increasingly induced in NB20, P20, NB20/P20, and NBP20 cells (NB20<P20< NB20/P20< NBP20). Accordingly, cumulative co-exposures of cells to NBP resulted in acquisition of significantly increased stem-like cell populations that should be considered a novel cancer-associated property used to measure the progression of breast cell carcinogenesis.

The ability to develop stem-like cells is associated with activation of the EMT program [37]. During EMT, reduction of epithelial cellular adhesion molecule (EpCAM) and E-cadherin is associated with a loss in cell-cell adhesion [42] while an increase in matrix metalloproteinase-9 (MMP-9) is involved in degradation of the extracellular matrix [37]; increased Vimentin plays a role in filament formation and cell motility [43]. We detected that EpCAM and E-cadherin were reduced but MMP-9 and Vimentin were increased (Figure 2C) in NB- and/or PhIP-exposed cells, and these changes occurred consistently with degrees of increased mammosphere formation (2A) and enriched ALDH-positive stem-like cell populations (2B). Induction of the EMT program has been postulated to not only contribute to cellular acquisition of stem-like properties but also increased migratory and invasive capabilities [37]. We detected that increased degrees of cell migration (Figure 2D) and invasion (2E) were closely correlated with the increased degrees of mammosphere formation (2A), stem-like cell population (2B), and EMT markers (2C) acquired by NB- and/or PhIP-exposed cells: NBP20> NB20/P20>P20> NB20. In addition, using a wound healing assay, we detected that increased cell motility (Figure 2F-1 and 2F-2) was also accordingly acquired by these NB- and/or PhIP-exposed cells. These results indicate that cumulative exposure to NBP significantly enhanced cellular acquisition of stem-like and EMT-associated markers and properties. Cellular acquisition of stem-like and EMT-associated properties should be considered as novel cancer-associated properties and constitutive endpoints in measurement of the progression of breast cell carcinogenesis.

### NBP enhanced cancer-associated properties in breast cancer MCF7 cells

**Figure 3:**
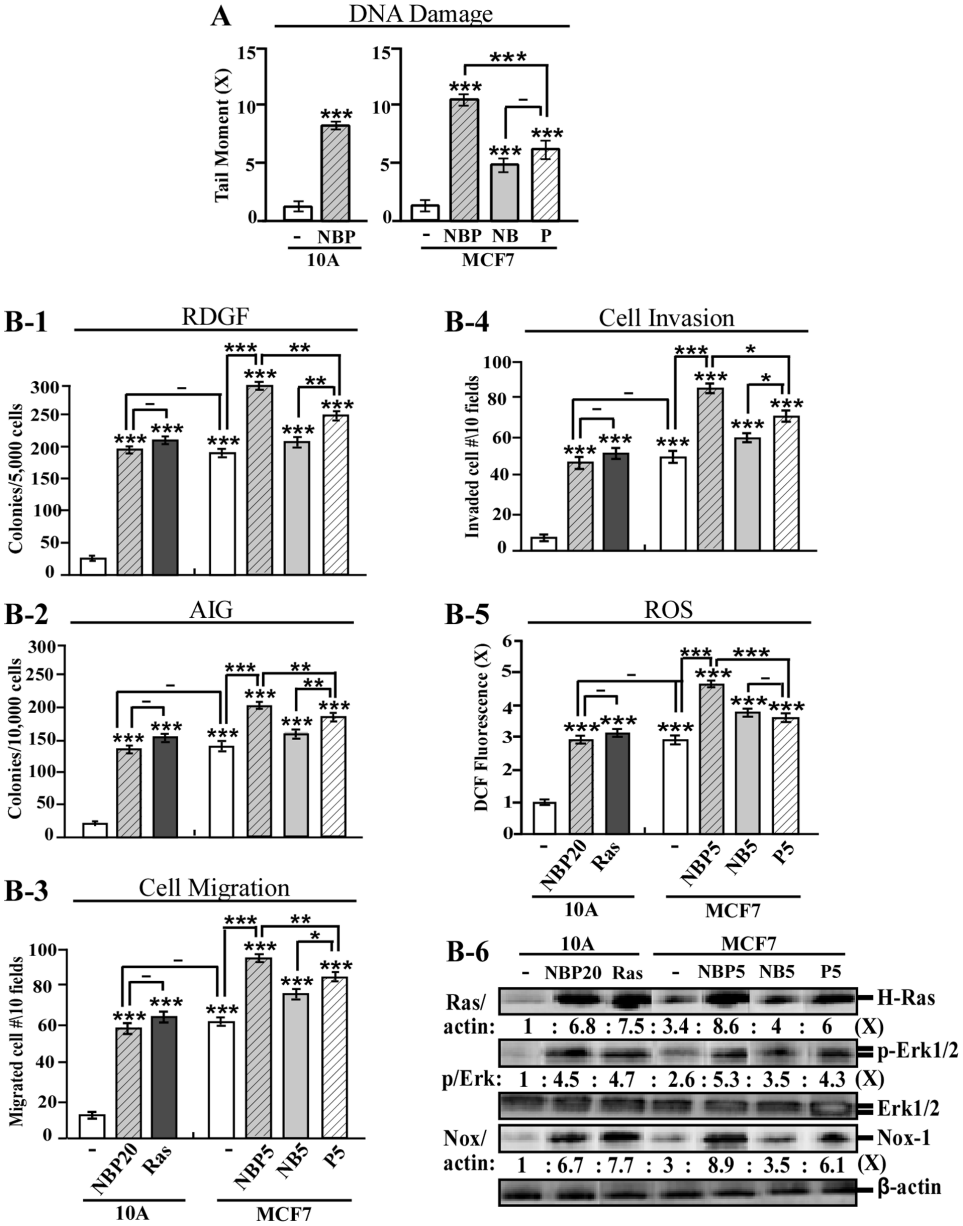
NBP-enhanced cancer-associated properties in breast cancer MCF7 cells. (A) MCF10A cells (10A) were treated with NBP, and MCF7 cells were treated with NBP, NB, or PhIP (P) for 24 h. DNA damage was measured by a comet assay and normalized by the value of average tail moment determined in untreated counterpart cells, set as 1 (X, arbitrary unit). (B-1 to B-6) MCF7 cells were exposed to NBP, NB, or PhIP for five cycles (NBP5, NB5, and P5). The NBP20 and MCF10A-Ras (Ras) cell lines were used as comparisons. (B-1) To determine cellular acquisition of RDGF, cells were maintained in LM medium for 10 days. Cell colonies ≥0.5 mm diameter were counted. (B-2) To determine cellular acquisition of AIG, cells were seeded in soft agar for 14 days. Cell colonies ≥0.1 mm diameter were counted. Cellular migratory (B-3) and invasive (B-4) activities were determined by counting the numbers of cells translocated through a polycarbonate filter without or with coated Matrigel, respectively, in 10 arbitrary visual fields. (B-5) Relative level of ROS as fold induction (X, arbitrary unit) was normalized by the level determined in untreated cells, set as 1. (B-6) Cell lysates were analyzed by immunoblotting using specific antibodies to detect levels of H-Ras, p-Erk1/2, Erk1/2, and Nox-1, with β-actin as a control, and these levels were quantified by densitometry. Levels of H-Ras (Ras/actin) and Nox-1 (Nox/actin) were calculated by normalizing with the level of β-actin and the level set in untreated control cells as 1 (X, arbitrary unit). Levels of specific phosphorylation of Erk1/2 (p/Erk) were calculated by normalizing the levels of p-Erk1/2 with the levels of Erk1/2, then the level set in control cells as 1 (X, arbitrary unit). Columns, mean of triplicates; bars, SD. All results are representative of three independent experiments. Statistical significance is indicated by * *P*<0.05, ** *P*<0.01, and *** *P*<0.001.

To clarify whether the enhanced acquisition of cancer-associated properties induced by NBP was limited to estrogen receptor (ER)-negative MCF10A cells, we exposed ER-positive human breast cancer MCF7 cells to NBP, NB, and PhIP. A single exposure to NBP induced more DNA damage in MCF7 cells than either NB or PhIP (Figure 3A), indicating NBP is more potent than either NB or PhIP alone to induce DNA damage for cellular carcinogenesis, even in cancer cells. After five cycles of carcinogen exposure, we detected significantly increased degrees of the cancer-associated properties of RDGF (Figure 3B-1), AIG (3B-2), cell migration (3B-3), and invasion (3B-4) acquired by carcinogen-exposed MCF7 cells versus parental cells. Although parental MCF7 cells possessed levels of these cancer-associated properties comparable to levels in NBP20 and MCF10A-Ras cells, these properties were significantly increased by NBP exposure, indicating that cumulative exposures to NBP may increase the potency of already cancerous cells. NBP was more potent than NB or PhIP in MCF7 cells, which is consistent with results seen in MCF10A cells. We next investigated if cumulative exposures to NBP, NB, and PhIP also resulted in induction of the biochemical endpoints of ROS elevation and Ras-Erk-Nox pathway activation in MCF7 cells as they did in MCF10A cells. We detected that parental MCF7 cells possessed higher levels of ROS (Figure 3B-5), H-Ras protein expression, Erk activation, and Nox-1 protein expression (3B-6) than MCF10A cells, and cumulative exposures to NBP were able to furthermore increase ROS production and Ras-Erk-Nox pathway induction in MCF7 cells. Exposure to NBP resulted in higher levels of ROS production (Figure 3B-5) and Ras-Erk-Nox pathway induction (3B-6) in MCF7 cells than either NB or PhIP alone, consistent with the higher levels of cancer-associated properties (3B-1 to B-4) acquired by NBP-exposed versus NB- or PhIP-exposed MCF7 cells. These results indicated that cumulative exposures to combined NNK, B[a]P, and PhIP were able to constitutively induce cellular acquisition of cancer-associated properties and associated biochemical endpoints in not only non-cancerous ER-negative MCF10A cells, but also cancerous ER-positive MCF7 cells in a similar manner. A combination of multiple carcinogens was more potent than individual carcinogens in long-term induction of breast cell carcinogenesis progression in both non-cancerous and cancerous cells in an ER-independent manner. Thus, co-exposure to low doses of NNK, B[a]P, and PhIP should be seriously considered in epidemiological studies to reveal the value of these carcinogens in the development of sporadic breast cancer. Given their ability to increase cancer-associated properties in non-cancerous or cancerous cells alike, it is important to identify agents capable of blocking breast cell carcinogenesis induced by cumulative exposures to combined carcinogens for early intervention of this disease.

### Intervention of NBP-induced carcinogenesis

In our previous reports, we demonstrated that GTCs, at non-cytotoxic levels, were capable of suppressing breast cell carcinogenesis induced by NB or PhIP [33]–[35]. EGCG, the major catechin present in green tea extract, is the most commonly studied preventive GTC [44], but some studies have shown that ECG may be more effective than EGCG in intervention of carcinogenesis [34], [45], [46]. ECG and EGCG have similar molecular masses, 442.37 and 458.37 g/mol, respectively. Our previous studies revealed that both ECG and EGCG at 40 µg/mL are toxic to MCF10A cells [34]. However, at a non-cytotoxic dose of 10 µg/mL, ECG is more effective than EGCG in suppression of NB-induced cellular carcinogenesis [34]. ECG at 10 µg/mL and EGCG at 5 µg/mL show comparable effectiveness in suppression of PhIP-induced cellular carcinogenesis [35]. Thus, to address whether ECG, EGCG, or a combination of both would be most effective in suppressing NBP-induced cellular carcinogenesis, we initially studied the effectiveness of ECG and EGCG, at non-cytotoxic doses, in blocking transient endpoints induced by a single exposure to NBP and subsequently verified their ability to suppress NBP-induced constitutive endpoints after cumulative exposures.

**Figure 4:**
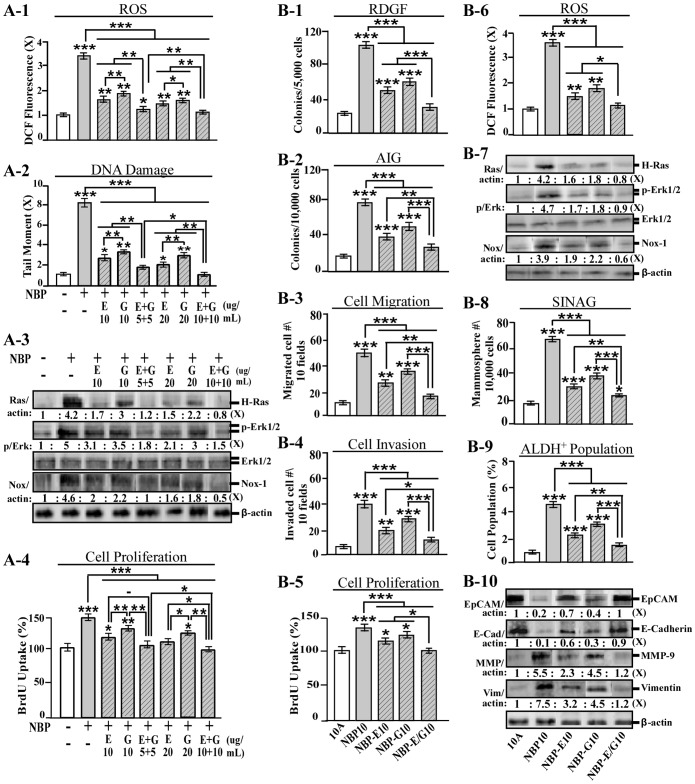
Intervention of NBP-induced carcinogenesis. (A-1 to A-4) MCF10A cells were treated with NBP in the absence and presence of ECG (E), EGCG (G), or a combination of ECG and EGCG (E+G) for 24 h. (B-1 to B-10) MCF10A (10A) cells were exposed to NBP in the absence and presence of 20 µg/mL ECG, 20 µg/mL EGCG, or combined 10 µg/mL ECG and 10 µg/mL EGCG (E/G) for 10 cycles, resulting in the NBP10, NBP-E10, NBP-G10, and NBP-E/G10 cell lines, respectively. (A-1 and B-6) Relative level of ROS as fold induction (X, arbitrary unit) was normalized by the level determined in untreated cells, set as 1. (A-2) Relative DNA damage was measured by a comet assay and normalized by the value of average tail moment determined in untreated counterpart cells, set as 1 (X, arbitrary unit). (A-3 and B-7) Cell lysates were analyzed by immunoblotting using specific antibodies to detect levels of H-Ras, phosphorylated-Erk1/2 (p-Erk1/2), Erk1/2, and Nox-1, with β-actin as a control, and these levels were quantified by densitometry. Levels of H-Ras and Nox-1 were calculated by normalizing with the level of β-actin and the level set in untreated control cells as 1 (X, arbitrary unit). Levels of specific phosphorylation of Erk1/2 (p/Erk) were calculated by normalizing the levels of p-Erk1/2 with the levels of Erk1/2, then the level set in control cells as 1 (X, arbitrary unit). (A-4 and B-5) Relative cell proliferation was determined and normalized by the value of BrdU detected in untreated cells, set as 100%. (B-1) To determine cellular acquisition of RDGF, cells were maintained in LM medium for 10 days. Cell colonies ≥0.5 mm diameter were counted. (B-2) To determine cellular acquisition of AIG, cells were seeded in soft agar for 14 days. Cell colonies ≥0.1 mm diameter were counted. (B-3) Cellular migratory and (B-4) invasive activities were determined by counting the numbers of cells translocated through a polycarbonate filter without or with coated Matrigel, respectively, in 10 arbitrary visual fields. (B-8) To determine cellular acquisition of the ability of serum-independent non-adherent growth (SINAG), cells were seeded in non-adherent cultures for 10 days; then, mammospheres (≥0.1 mm diameter) were counted. (B-9) Mammospheres were collected and trypsinized, and ALDH-expressing (ALDH^+^) cell population (%) was measured by flow cytometry. (B-10) Cell lysates were analyzed by immunoblotting using specific antibodies to detect levels of EpCAM, E-cadherin, MMP-9 and Vimentin, with β-actin as a control, and these levels were quantified by densitometry. The levels of EpCAM, E-cadherin, MMP-9 and Vimentin were calculated by normalizing with the level of β-actin and the level set in untreated control cells as 1 (X, arbitrary unit). Columns, mean of triplicates; bars, SD. All results are representative of three independent experiments. Statistical significance is indicated by * *P*<0.05, ** *P*<0.01, and *** *P*<0.001.

As shown in Figure 4, both ECG (E) and EGCG (G), at 10 or 20 µg/mL, were able to block NBP-induced transient endpoints of ROS elevation (4A-1) and DNA damage (4A-2), with ECG being more effective than EGCG in suppressing these properties. Interestingly, a combination of ECG and EGCG (E+G) at 5 or 10 µg/mL each was more effective than individual catechins at 10 or 20 µg/mL, respectively, in suppression of NBP-induced ROS and DNA damage, indicating that combined ECG and EGCG were optimal for blocking ROS elevation and DNA damage induced by combined NNK, B[a]P, and PhIP. Consistently, ECG was more effective than EGCG, and a combination of ECG and EGCG was more effective than individual catechins in suppression of Ras expression, Erk activity, and Nox-1 expression (Figure 4A-3), as well as cellular proliferation (4A-4) transiently induced by a single exposure to NBP. These results indicate that a combination of ECG and EGCG, at non-cytotoxic levels, was more effective than individual agents in blocking NBP-induced transient endpoints, which are essential for induction of cellular carcinogenesis.

To verify the ability of ECG and EGCG to intervene in the acquisition of NBP-induced cancer-associated properties, we repeatedly exposed MCF10A cells to NBP in the absence or presence of 20 µg/mL ECG (E), 20 µg/mL EGCG (G), or combined 10 µg/mL ECG and 10 µg/mL EGCG (E/G) for 10 cycles, resulting in the NBP10, NBP-E10, NBP-G10, and NBP-E/G10 cell lines, respectively. We detected that ECG was more effective than EGCG and a combination of ECG and EGCG was more effective than individual catechins in intervention of cellular acquisition of RDGF (Figure 4B-1), AIG (4B-2), increased cell migration (4B-3), invasion (4B-4), and proliferation (4B-5), as well as ROS elevation (4B-6) and Ras-Erk-Nox pathway activation (4B-7). Furthermore, NBP-increased mammosphere formation (Figure 4B-8) and ALDH-positive stem-like cell population (4B-9) as well as EMT-associated MMP-9 induction, Vimentin induction, EpCAM reduction, and E-cadherin reduction (4B-10) were also blocked by co-exposure to ECG and EGCG. Consistently, ECG was more effective than EGCG, and a combination of ECG and EGCG was more effective than individual catechins in suppressing these properties. These results indicated that ECG and EGCG, at non-cytotoxic levels, were effective in suppression of breast cell carcinogenesis associated with long-term exposure to NBP. A combination of ECG and EGCG was more effective than either agent alone, at equivalent doses, in suppression of NBP-induced transient endpoints and constitutive cancer-associated properties. However, whether non-cytotoxic ECG and EGCG are effective in prevention of sporadic breast cancer development associated with long-term exposure to NBP will need epidemiological and clinical studies for validation.

Our unique model presents several advantages over others to advance our understanding of human breast carcinogenesis and early intervention. It is highly sensitive in detecting physiologically-achievable, low doses of either weak or potent carcinogens capable of inducing breast cell carcinogenesis. It takes a unique approach of exposing cells to carcinogens in a cumulative manner for progressive induction of chronic carcinogenesis from non-cancerous to pre-cancerous and cancerous stages. In addition, it uses measurable transient and constitutive cellular, biochemical, and molecular endpoints to determine the induction and progression of cellular carcinogenesis and to identify non-cytotoxic agents effective in intervention. In this communication, we used our model to demonstrate, for the first time, the ability of two relatively weak breast carcinogens, NNK and B[a]P, to enhance breast cell carcinogenesis induced by potent breast carcinogen PhIP; we also identified the ability of combined ECG and EGCG, which was more effective than individual catechins, to intervene in carcinogenesis induced by combined NNK, B[a]P, and PhIP. We reported that transient induction of ROS, the Ras-Erk-Nox pathway, increased cell proliferation, and DNA damage accounted for the mechanisms of initiation of cellular carcinogenesis during each exposure to NNK, B[a]P, and/or PhIP [34], [35], ultimately leading to acquisition of cancer-associated properties and progression of cellular carcinogenesis induced by cumulative exposures. These transient endpoints were highly enhanced by combined NNK, B[a]P, and PhIP. Blockage of ROS elevation or the Ras-Erk-Nox pathway significantly reduced NBP-induced DNA damage and cell proliferation, indicating essential roles of ROS elevation and Ras-Erk-Nox pathway activation in NBP-induced cellular carcinogenesis. Accordingly, transient induction of ROS elevation and the Ras-Erk-Nox pathway served not only as markers for detecting NBP effects but also as targets for suppressing NBP-induced carcinogenesis. As we demonstrated in this communication, we initially used ROS elevation and the Ras-Erk-Nox pathway as targets to detect the optimal concentration and combination of ECG and EGCG effective in suppression of NBP-induced transient endpoints. Subsequently, we used cancer-associated properties/constitutive endpoints as targets to verify the effectiveness of combined ECG and EGCG in intervention of NBP-induced progression of cellular carcinogenesis. Previously, we reported the mitogenic ability of NNK at 100 pmol/L, B[a]P at 100 pmol/L, and PhIP at 10 nmol/L to induce increased cell proliferation in a single exposure and after cumulative exposures [29]–[36]. Here, we showed that a combination of these carcinogens (NBP) at these same doses was also mitogenic (Figures 1B-4, 4A-4, &4B-5). In addition, induction of reduced viability or cell death by NBP was not detectable (data not shown), indicating that NBP was not cytotoxic at all. Thus, like NB- or PhIP-induced carcinogenesis progression [33]–[36], NBP-induced breast cell carcinogenesis did not involve the selection of apoptosis-resistant carcinogenic clones; instead, cumulative exposures to NBP resulted in progressive cellular acquisition of cancer-associated properties.

In addition, our model system is able to address whether preventive agents are effective in intervention of cancer stem-like cell development. Development of cancer stem-like cells, involving induction of the EMT program, has been postulated to play important roles in cancer development [37] and cancer recurrence after chemotherapy [47]. Using our model, we demonstrated the ability of NBP to induce increased stem-like cell population and the EMT program in ER-negative MCF10A cells (Figures 2A–F, 4B-3, 4B-4, and 4B-8 to 4B-10). These results provide evidence for the involvement of mesenchymal stem-like properties during the carcinogenic transformation of ER-negative breast cells. In addition, long-term NBP exposure resulted in acquisition of increased cancer-associated properties by not only non-cancerous MCF10A but also cancerous MCF7 cells in an ER-independent manner. Possibly, long-term exposure to NBP may also result in increased mesenchymal stem-like properties in other types of breast cancer cells, such as basal-like, triple-negative cancer cells. However, whether long-term exposure of triple-negative cancer cells to NBP will result in increased carcinogenic potency remains to be clarified. Using our model, we also demonstrated that combined ECG and EGCG was more effective than individual agents in intervention of NBP-increased stem-like cell population and EMT program induction. Thus, applying our model will help accelerate our understanding of low-dose carcinogens in breast cell carcinogenesis and identification of preventive agents effective in reducing the health risk of sporadic breast cancer associated with chronic exposure to low doses of environmental and dietary carcinogens.
